# Contrast Sensitivity in Microtropic and Anisometropic Eyes of Successfully Treated Amblyopes

**DOI:** 10.4274/tjo.52261

**Published:** 2017-04-01

**Authors:** Özlem Öner, Sezin Akça Bayar, Sibel Oto, Onur Gökmen, Mustafa Agah Tekindal

**Affiliations:** 1 Söke Fehime Faik Kocagöz Hospital, Ophthalmology Clinic, Aydın, Turkey; 2 Başkent University Faculty of Medicine, Department of Ophthalmology, Ankara, Turkey; 3 Başkent University Faculty of Medicine, Department of Biostatistics, Ankara, Turkey

**Keywords:** contrast sensitivity, amblyopia, microtropia, Anisometropia

## Abstract

**Objectives:**

To assess and compare contrast sensitivity function in the previously amblyopic and non-amblyopic “normal” eyes of patients with microtropia and anisometropia who achieved 20/20 visual acuity after occlusion therapy.

**Materials and Methods:**

Contrast sensitivity was tested monocularly on both eyes of 34 successfully treated microtropic and 15 anisometropic subjects (visual acuity 20/20 in both eyes). Contrast sensitivity function was evaluated by CSV-1000E and age-matched nomograms were used (spatial frequencies of 3, 6, 12, and 18 cycles per degree [cpd]) for comparison.

**Results:**

The mean age of subjects was 11.2±1.3 years in the microtropic group, 9.8±1.7 years in the anisometropic group (7-12 years); the mean follow-up time was 16.4±3.2 months (12 to 92) in the microtropic group and 27.7±1.8 months (12-84) in the anisometropic group. Statistical comparison of the microtropic amblyopic eyes versus non-microtropic eyes showed significant differences at spatial frequencies of 3, 12 and 18 cpd (3 cpd, t=2.8, p=0.007; 6 cpd, t=1.1 p=0.261; 12 cpd, t=2.2, p=0.033; 18 cpd, t=2.2, p=0.030). When anisometropic eyes were compared with non-anisometropic eyes, there was a significant difference only at 12 cpd (t=2.1 p=0.049). The comparison of non-amblyopic eyes versus age-matched nomograms revealed no differences at any of the spatial frequencies (p>0.05 for all).

**Conclusion:**

Contrast sensitivity was decreased in patients with amblyopia, especially in the microtropic group. The assessment of contrast sensitivity function may serve as a new parameter for termination of occlusion therapy.

## INTRODUCTION

Amblyopia, which occurs in 2-4% of the population,^1,2,3^ is a developmental visual disorder resulting in reduced visual acuity in one eye due to strabismus, anisometropia, or deprivation in early childhood.^2,3,4,5,6,7,8^ The main sign of amblyopia is the presence of decreased vision in one or both eyes without any identifiable ocular pathology. This reduction in visual acuity cannot be improved with refractive correction.^3,5,9^

Although amblyopia is usually diagnosed as a decrease in vision in a single eye, amblyopes also suffer widespread deficits in spatial function. When quantifying variations in the vision systems of amblyopes, most of these deficits can be reduced to two basic visual parameters, visual acuity and contrast sensitivity.^1,2,4,5,6,10^

Contrast sensitivity function (CSF) is the ability to distinguish sinusoidal gratings within a range of spatial frequencies.^4,9,10^ Contrast sensitivity^10^ and spatial localization^9,11^ are reduced in amblyopia due to developmental defects in the spatial visual processes of the nervous system. The effect of occlusion therapy (patching the stronger eye) in amblyopic patients on CSF is controversial.^9^ Although visual acuity is a conventional evaluation used in the treatment of amblyopia and assesses the spatial resolution limits of vision, it cannot predict an individual’s performance in other spatial vision tasks such as target perception or discrimination. Therefore, it has been proposed that CSF is a better tool for diagnosing and investigating spatial visual deficits.^4^

The aim of this study was to evaluate differences in contrast sensitivity between the amblyopic and normal eyes of patients with microtropia and anisometropia that were adequately rehabilitated with occlusion therapy.

## MATERIALS AND METHODS

### Patients

Thirty-four microtropic and 15 anisometropic patients were retrospectively included in the study. The contrast sensitivity test was performed on each eye separately by one of the authors (Ö.Ö.), who was blinded to the patients’ clinical condition. After informed consent forms were obtained from the patients’ families, the patients underwent ophthalmic and orthoptic examinations. Inclusion criteria were: 1) age between 7 and 12 years old, because the minimum age that allowed for reliable contrast sensitivity test with the CSV-1000E (VectorVision; Dayton, OH, USA) was 7 years old; 2) the presence of congenital, stable fixation not indicating latent or manifest nystagmus on clinical examination; 3) visual acuity of 20/20 or better in the amblyopic and nonamblyopic eyes; 4) history of at least 1 year of successful occlusion therapy; 5) no history of previous surgery; and 6) correction of refractive errors prior to the contrast sensitivity test. Additional criteria for microtropic patients were: 1) deviation less than 10 prism diopters in the absence of alternation; and 2) anisometropia less than 1.5 diopters. Patients were evaluated with their best corrected eyeglass prescription. Snellen decimal charts were used to assess visual acuity.

As amblyopia treatment, patients’ nonamblyopic eyes were covered with standard opaque patches. The duration of occlusion therapy was determined based on the patient’s age, and the type and severity of amblyopia.

### Contrast Sensitivity Test

Contrast sensitivity data were obtained using the CSV-1000E (VectorVision, Dayton, OH, USA) contrast sensitivity instrument, which consists of a rear-illuminated translucent chart that automatically calibrates to a dim light level of 85 candela per meter squared (cd/m2). The chart consists of vertical sinewave gratings at 4 spatial frequencies (3, 6, 12, and 18 cycles per degree [cpd]), each shown on a separate row.

Each row contains 8 pairs of circular patches, one of which contains the sinewave grating while the other is blank. Each of the 4 spatial frequencies are presented at 8 different contrast levels: 3 cpd (range, 0.70-2.08 log units), 6 cpd (range, 0.91-2.29 log units), 12 cpd (range, 0.61-1.99 log units), and 18 cpd (range, 0.17-1.55 log units). The contrast level in each row decreases from left to right by 0.17 log units between patches 1 through 3, and by 0.15 log units between patches 3 through 8.

**Figure 1:**
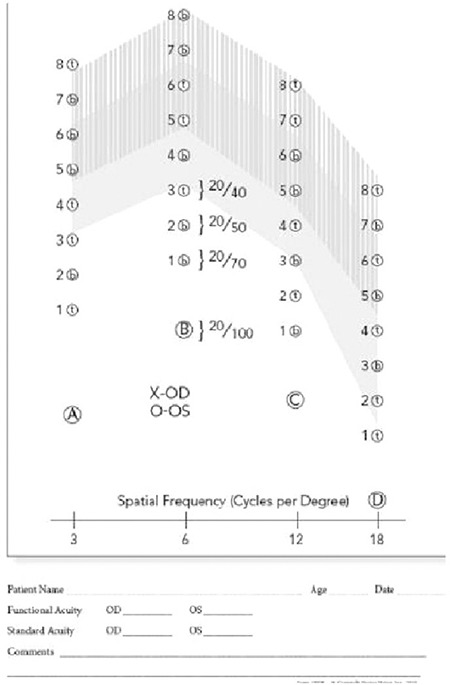
CSV-1000E contrast sensitivity form

The tests were performed monocularly using best refractive correction without pupil dilation from a distance of 3 m using the CSV-1000E contrast chart test face (VectorVision). The eye not being evaluated was covered during the test. The 4 spatial frequencies (3, 6, 12, and 18 cpd) were tested using a two-alternative mandatory selection procedure without orientation. The patients were first asked whether there was a test grating in the presented pairs of stimulus patches, and if yes, whether the grating was in the top or bottom patch of each pair. The test was repeated twice, and the last correct response for each row was accepted as the contrast threshold for the corresponding spatial frequency. These thresholds were recorded on the special diagram that accompanies the CSV-1000E. The diagram’s horizontal axis represents spatial frequency (3, 6, 12, and 18 cpd), and the vertical axis represents the contrast level in logarithmic units. Marking the contrast threshold for each spatial frequency creates the contrast sensitivity curve (Figure 1).

### Statistical Analysis

Statistical analyses of logarithmic unit values were done using paired-samples t-test and Wilcoxon signed-rank test with the SPSS statistical software package (SPSS Inc., Chicago, IL, USA). P values less than 0.05 were accepted as statistically significant. For all spatial frequencies, the amblyopic eyes were compared to non-amblyopic fellow eyes and non-amblyopic eyes were compared to age-matched nomograms. Furthermore, microtropic and anisometropic eyes were compared to determine whether amblyopia type has an effect on contrast sensitivity.

## RESULTS

Mean ages were 11.2±1.3 years for the microtropic groups and 9.8±1.7 years for the anisometropic group (range, 7-12 years for both groups). Mean follow-up time was 16.4±3.2 (range, 12-92 months) for the microtropic group and 27.7±1.8 (range, 12-84 months) for the anisometropic group. There was a significant difference between the microtropic group and the anisometropic group in duration of occlusion therapy (12.9 and 35 months, respectively, p=0.010). The age at initiation of occlusion therapy was 9.6±2.2 years in the microtropic group and 7.3±2.9 years for the anisometropic group, a statistically significant difference (t=2.4, p=0.026) (Table 1).

**Figure 2:**
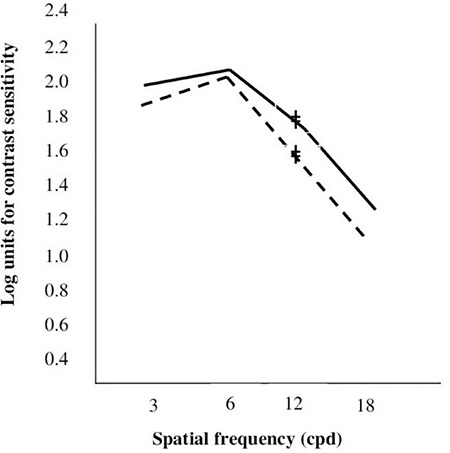
Mean contrast sensitivity function values of the microtropic eyes (dotted line) and non-microtropic eyes (solid line)

**Figure 3:**
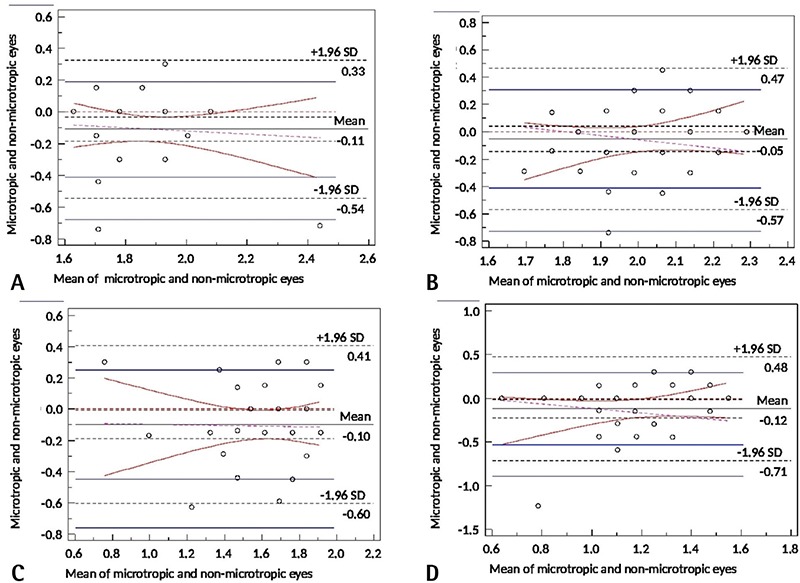
Bland-Altman plot of the microtropic group at spatial frequencies of 3 cpd (A), 6 cpd (B), 12 cpd (C), and 18 cpd (D) SD: Standard deviation

**Figure 4:**
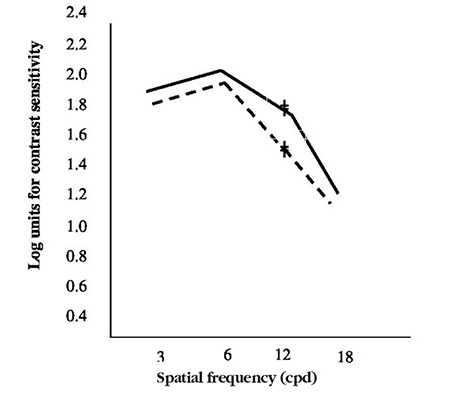
Mean contrast sensitivity function values of the anisometropic eyes (dotted line) and non-anisometropic eyes (solid line)

**Figure 5:**
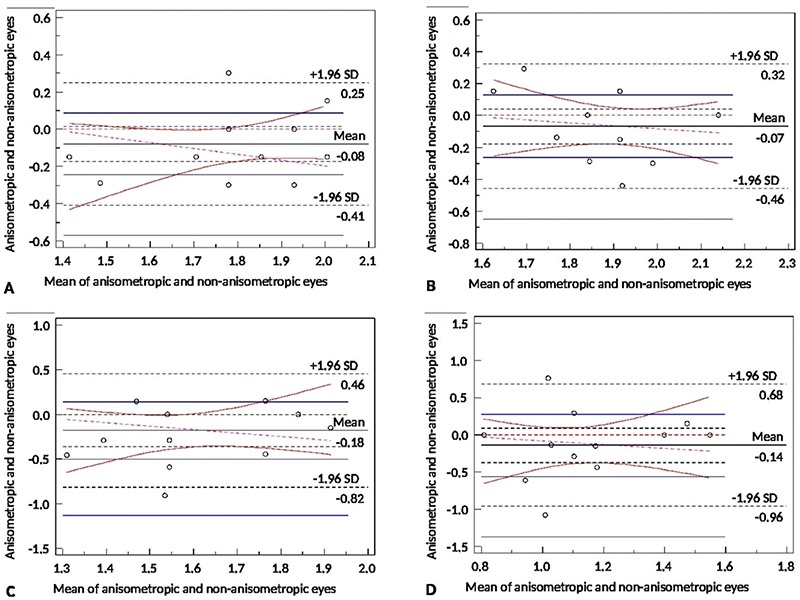
Bland-Altman plot of the anisometropic group at spatial frequencies of 3 cpd (A), 6 cpd (B), 12 cpd (C), and 18 cpd (D) SD: Standard deviation

Paired-samples test of microtropic eyes and non-microtropic eyes showed significant differences at spatial frequencies of 3, 12, and 18 cpd (3 cpd: t=2.8, p=0.007; 12 cpd: t=2.2, p=0.033; 18 cpd: t=2.2, p=0.030) (Figure 2, Figure 3A, 3B, 3C, 3D), but there was no significant difference at 6 cpd (6 cpd, t=1.1 p=0.261). In paired t-test of the anisometropic and non-anisometropic eyes, a slight reduction was observed at 12 cpd, while no significant differences emerged in 3, 6, or 18 cpd (3 cpd, t=1.8 p=0.089; 6 cpd, t=1.3 p=0.207; 18 cpd, t=1.2 p=0.219) (Figure 4, Figure 5A, 5B, 5C, 5D). Comparison of non-microtropic and non-anisometropic eyes with age-matched nomograms revealed no significant differences at any of the spatial frequencies (non-microtropic eyes: 3 cpd, p=0.075; 6 cpd, p=0.670; 12 cpd, p=0.846; 18 cpd, p=0.121; non-anisometropic eyes: 3 cpd, p=0.454; 6 cpd, p=0.116; 12 cpd, p=0.309; 18 cpd, p=0.196).

**Figure 6:**
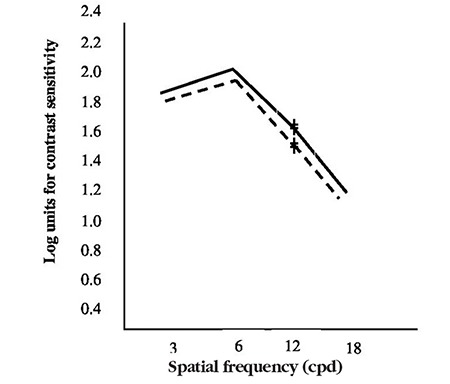
Mean contrast sensitivity function values of the anisometropic eyes (dotted line) and microtropic eyes (solid line)

No statistical differences were found using paired t-test between the CSFs of the 34 microtropic patients and the 15 anisometropic patients at any spatial frequency (3 cpd, t=1.1 p=0.254; 6 cpd, t=2.0 p=0.057; 12 cpd, t=1.7 p=0.103; 18 cpd, t=0.8 p=0.418) (Figure 6).

As shown in Table 2, best corrected visual acuities of the microtropic and anisometropic groups were 0.33 and 0.25 logMAR, respectively, but the difference was not statistically significant (t=0.80, p=0.435). Both groups showed statistically significant improvements after treatment (microtropic group, 0.00 logMAR; anisometropic group, -0.01 logMAR; t=1.46, p=0.164).

Randot values before occlusion therapy were 20.6% positive in the microtropic group and 40.0% positive in the anisometropic group; after therapy, these values increased to 41.2% in the microtropic group and 66.7% in the anisometropic group (Table 2).

## DISCUSSION

This study focused on the contrast sensitivity values of eyes that became amblyopic due to microtropia or anisometropia and were later successfully rehabilitated using occlusion therapy. We observed significant differences between microtropic and non-microtropic eyes at spatial frequencies of 3, 12, and 18 cpd. Despite regaining normal visual acuity after occlusion therapy, microtropic eyes still exhibited reduced CSF compared to non-microtropic eyes. There was also a significant difference between anisometropic and non-anisometropic eyes at 12 cpd. The literature yields contradictory results on this topic. Some studies have reported that contrast sensitivity levels in amblyopia are normal or near normal at low spatial frequencies and decreased at high spatial frequencies.^8,9,12,13,14^ However, while some of the more recent contrast sensitivity studies have detected deficits in amblyopes only at high spatial frequencies, others have demonstrated reductions at all spatial frequencies.^15,16^ The normal contrast sensitivity curve peaks at a spatial frequency of 5-6 cpd.^11^ In the present study, there was no statistical difference between amblyopic eyes and normal eyes at 6 cpd. These differences may be related to differences in the instruments or contrast sensitivity test methods used.

Previous studies have reported that contrast sensitivity levels are reduced at high spatial frequencies in the amblyopic eye of patients with amblyopia,^17,18,19^ but normal or near normal at low spatial frequencies (less than 6 cpd). Chatzistefanou et al.^9^ also found that the normal eyes of amblyopic patients had abnormal CSF, regardless of whether they had undergone occlusion therapy. In contrast, Zele et al.^20^ found that the normal eyes of amblyopes had normal values at all spatial frequencies. Moreover, Maebera et al.^8^ reported that contrast sensitivity was reduced in amblyopic eyes, while normal eyes were generally normal. Similar to Zele et al.^20^, in the present study we detected no significant differences in CSF throughout the spatial frequency range when the normal eyes of microtropic and anisometropic patients were compared with age-matched nomograms.

There are a few theories which may explain the differences in contrast sensitivity values we observed between the anisometropic and non-anisometropic eyes and between the microtropic and non-microtropic eyes in this study. Firstly, the age at diagnosis and initiation of treatment was low among the anisometropic amblyopic children in this study. In addition, the duration of treatment was longer in the anisometropic group. Lai et al.^21^ reported that the vision system has greater plasticity in early childhood, which may be related to the difference in our results. Secondly, it has been suggested in the literature that in anisometropia, visual acuity is affected more than contrast sensitivity.^14^ In their most recent study, Tang et al.^22^ proposed that anisometropic amblyopes may have intact integration of motion information provided by moving component gratings. They attributed the apparent deficiencies in contrast sensitivity for moving plaids in anisometropic amblyopes almost entirely to these gratings, which are low-level processing deficits. Therefore, the difference observed in our study may be related to amblyopia type.

## CONCLUSION

In summary, contrast sensitivity assessment may provide valuable information regarding visual function in amblyopic patients, could guide occlusion therapy, and may be a new parameter in the termination of occlusion therapy.

## Figures and Tables

**Table 1:**
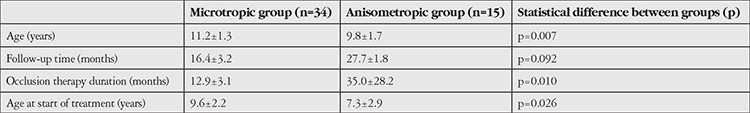
Statistical comparison of age, follow-up time, age at start of treatment, and duration of occlusion therapy in anisometropic and microtropic amblyopes

**Table 2:**
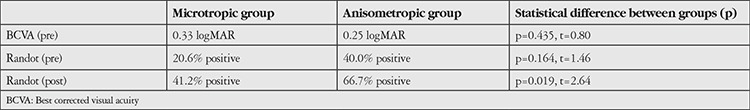
Statistical comparison of best corrected visual acuity and Randot values pre- and post-treatment in anisometropic and microtropic amblyopes
